# DFT data to relate calculated LUMO energy with experimental reduction potentials of Cu(II)-β-diketonato complexes

**DOI:** 10.1016/j.dib.2021.107331

**Published:** 2021-08-27

**Authors:** Marrigje M. Conradie, Ernst H.G. Langner, Jeanet Conradie

**Affiliations:** Department of Chemistry, Faculty of Natural and Agricultural Sciences, PO Box 339, University of the Free State, Bloemfontein, 9300, South Africa

**Keywords:** DFT, LUMO energy, Cu-β-diketonato complexes

## Abstract

We present data on the computed lowest unoccupied molecular orbital energy (*E_LUMO_*) of two series of Cu(II)-β-diketonato complexes, calculated via density functional theory (DFT). These are correlated to experimental reduction potential data (*E*_pc_), obtained by cyclic voltammetry under different experimental conditions (solvent, working and reference electrodes). All calculations were done with the B3LYP functional in the gas phase. Knowledge of the influence of different ligands on the redox potential of copper complexes, as measured by DFT calculated energy data, are very useful. These theoretical correlations are vital in the further design of similar compounds, to be customized for specific applications. The correlations can be used to predict and fine-tune redox potentials prior to synthesis, saving experimental chemists time and laboratory expenses. Redox potentials influence the catalytic property of bis(β-diketonato)copper(II) compounds. New catalysts can therefore be customized with a specific reduction potential and catalytic activity. Further, the Cu(II/I) redox couple is a potential alternative as electrolyte for dye-sensitized solar cells [1], [2], [3]. The redox potential of the electrolyte can drastically affect the photovoltage output and should therefore be optimized for efficiency and durability. By adjusting the reduction potential via different ligands on the complex, the properties of copper dyes can be fine-tuned at molecular level. For more insight into the reported data, see the related research article “Synthesis, Characterization, DFT and Biological Activity of Oligothiophene β-diketone and Cu-complexes” published in Polyhedron [4].

## Specifications Table

**Table:** 

Subject	Physical and Theoretical Chemistry
Specific subject area	DFT calculations of chemical structures.
Type of data	Table Graph Figure
How data were acquired	Electronic structure calculations, using the Gaussian 16 program
Data format	Raw analysed
Parameters for data collection	Geometry optimizations were done using the Gaussian 16 software program, in gas phase, using the B3LYP functional and the 6–311G(d,p) basis set.
Description of data collection	Data was collected from DFT output files
Data source location	University of the Free State Bloemfontein South Africa
Data accessibility	With the article
Related research article	N.G.S. Mateyise, S. Ghosh, M. Gryzenhout, E. Chiyindiko, M.M. Conradie, E.H.G. Langner, J. Conradie, Synthesis, characterization, DFT and biological activity of oligothiophene beta-diketone and Cu-complexes, Polyhedron. 205 (2021) 115290. https://doi.org/10.1016/j.poly.2021.115290

## Value of the Data

• The relationship between *E_LUMO_* and the experimental reduction potential *E*_pc_ of copper(II)-β-diketonato complexes is important in the field of catalysis, as it allows to predict their catalytic activity.
• *E*_pc_ vs *E*_LUMO_ relationships can be used by experimental chemists in the design of customized complexes with a desired reduction potential.
• Calculated LUMO energy data provides insight into the influence of different ligands on the redox potential of copper complexes.
• Redox potentials are important for researchers interested in copper electrolytes under low light conditions, for commercialization of dye sensitized solar cell (DSSC) technology.

## 1 Data Description

**Scheme 1:**
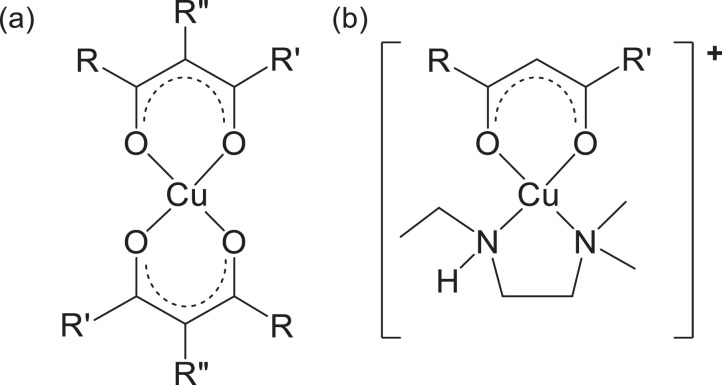
Copper(II)-β-diketonato compounds of this study: (a) [Cu^II^(β-diketonato)_2_] and (b) [Cu^II^(β-diketonato)(dmeen)]^+^ complexes, where dmeen = N,N-dimethyl-N'-ethyl-1,2-diaminoethane. Groups R, R” and R’ and complex numbering are indicated in Table 1.

**Fig. 1:**
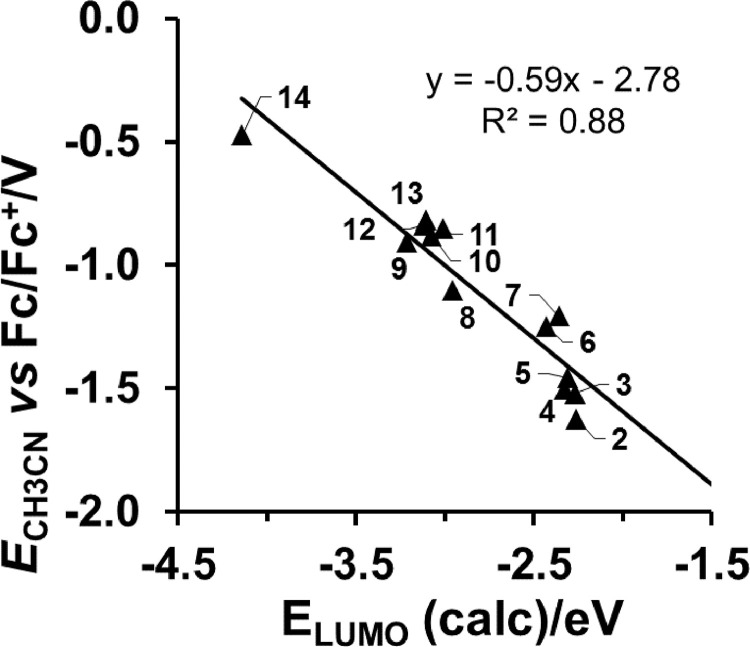
CH_3_CN as solvent and glassy carbon working electrode: Relationship between experimental reduction potentials *E*_pc_ (V *versus* Fc/Fc^+^) of [Cu^II^(β-diketonato)_2_] complexes (**2**) - (**14**), and their DFT calculated energy *E*_LUMO_. Data and complex numbering given in Table 1.

**Fig. 2:**
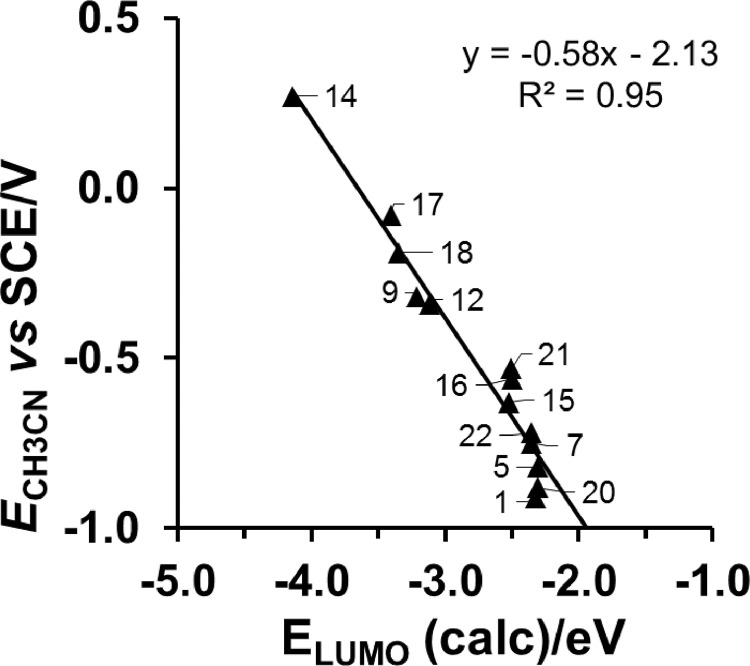
CH_3_CN as solvent and carbon fibres working electrode: Relationship between experimental reduction potentials *E*_pc_ (V *versus* SCE) of [Cu^II^(β-diketonato)_2_] complexes (**1**), (**5**), (**7**), (**9**), (**12**), (**14**), (**15**) – (**18**), (**20**) - (**22**), and their DFT calculated energy *E*_LUMO_. Data and complex numbering given in Table 1. Complex (**19**) did not fit the trend.

**Fig. 3:**
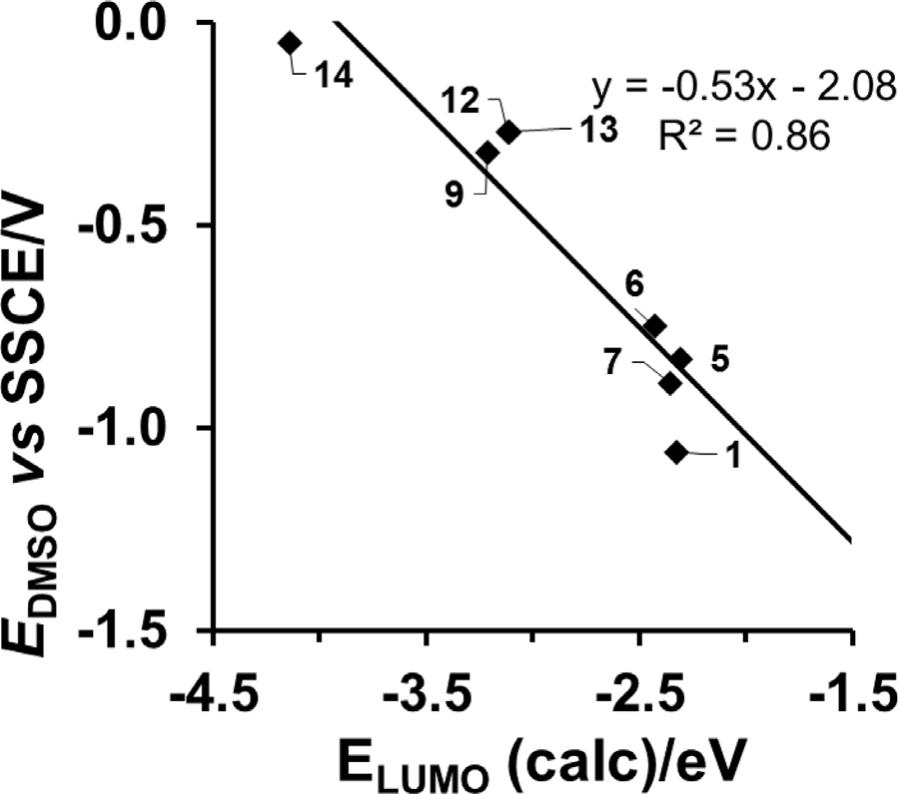
DMSO as solvent and carbon fibres working electrode: Relationship between experimental reduction potentials *E*_pc_ (V *versus* SSCE) of [Cu^II^(β-diketonato)_2_] complexes (**1**), (**5**) – (**7**), (**9**), (**12**) - (**14**), and their DFT calculated energy *E*_LUMO_. Data and complex numbering given in Table 1.

**Fig. 4:**
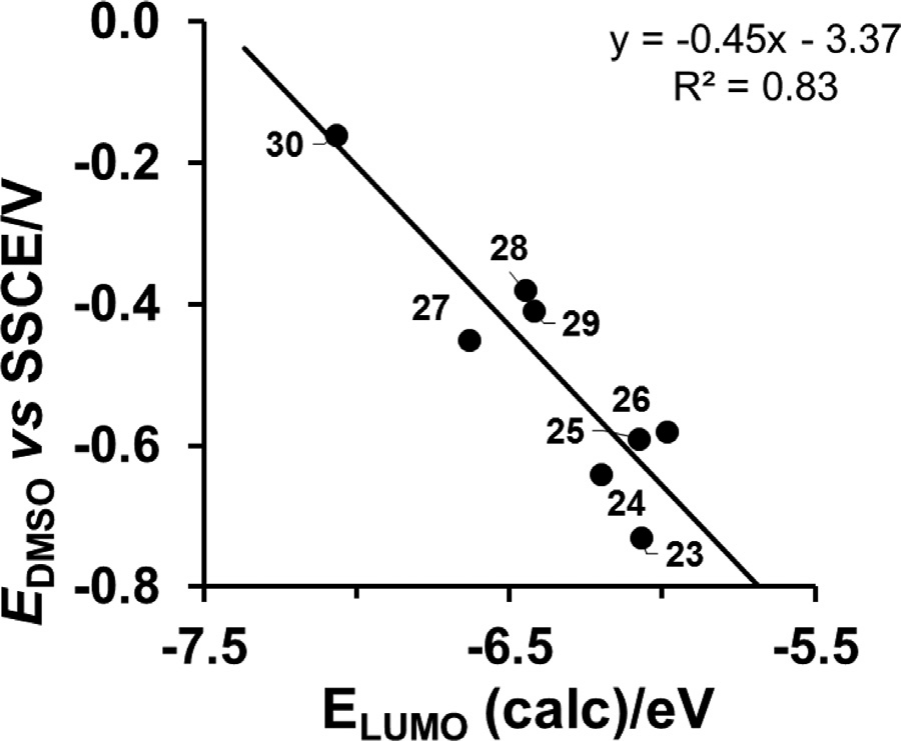
DMSO as solvent and carbon fibres working electrode: Relationship between experimental reduction potentials *E*_pc_ (V *versus* SSCE) of [Cu^II^(β-diketonato)(dmeen)]^+^ complexes (**23**) - (**30**), and their DFT calculated energy *E*_LUMO_. Data and complex numbering given in Table 1.

The complexes shown in Scheme 1 have been studied via DFT and their optimized Cartesian coordinates are given in the supporting information. *E*_LUMO_ data and complex numbering are listed in Table 1. Linear relationships between experimental *E*_pc_ and computed *E*_LUMO_ of the copper (II/I) reduction, are shown in Fig. 1, Fig. 1, Fig. 2, Fig. 3. These values were obtained for series of related molecules obtained under the same experimental conditions (solvent, working and reference electrodes). In Figs. 1 and 2, the *E*_pc_ *vs E*_LUMO_ relationships for two different series of [Cu^II^(β-diketonato)_2_] compounds are shown. For both these series, *E*_pc_ (Cu^II/I^) was obtained in the same solvent CH_3_CN, but with different working and reference electrodes [4], [5], [6], [7]. In Fig. 3 the *E*_pc_ *vs E*_LUMO_ relationship is shown only for [Cu^II^(β-diketonato)_2_] compounds, with *E*_pc_ (Cu^II/I^) obtained in DMSO as solvent [8]. Fig. 4 provides the *E*_pc_ *vs E*_LUMO_ relationship for [Cu^II^(β-diketonato)(dmeen)]^+^ compounds, with *E*_pc_ (Cu^II/I^) also obtained in DMSO as solvent [8]. Experimental *E*_pc_ data is reported either *versus* the redox couple of ferrocene (Fc/Fc^+^ [9]), or against the saturated calomel electrode (SCE) or the saturated salt calomel electrode (SSCE). The reference values used are either E (SCE) = 0.241 V or E (SSCE) = 0.2360 V, *versus* the normal hydrogen electrode (NHE). The catalytic property and application of copper-β-diketonato compounds as redox mediators for dye-sensitized solar cells (DSSC), depends on their redox potential [1], [2], [3],^10^,11]. This is why the theoretical prediction of the redox potential of copper-β-diketonato compounds from these existing *E*_pc_ - *E*_LUMO_ relationships, is indispensable.

**Table 1:** B3LYP/6–311G(d,p) calculated LUMO energies and experimental reduction potentials measured in different solvents and with different reference electrodes. Values are listed both for [Cu^II^(β-diketonato)_2_] compounds (**1**) – (**22**) and [Cu^II^(β-diketonato)(dmeen)]^+^ compounds (**23**) – (**30**), with β-diketonato = (RꞌCOCR”COR)^−^.

No	Rꞌ	R”	R	E_LUMO_ (eV)	*E*_pc_ (V) *vs* SSCE^a^	*E*_pc_ (V) *vs* SCE^b^	*E*_pc_ (V) *vs* Fc/Fc^+^^c^
[Cu^II^(β-diketonato)_2_]							

**1**	C(CH_3_)_3_	H	C(CH_3_)_3_	−2.33	−1.06	−0.91	
**2**	Fc d	H	CH_3_	−2.26			−1.624
**3**	Fc	H	Fc	−2.26			−1.520
**4**	Fc	H	Ph	−2.33			−1.503
**5**	CH_3_	H	CH_3_	−2.31	−0.83	−0.82	−1.458
**6**	Ph d	H	Ph	−2.43	−0.75		−1.247
**7**	Ph	H	CH_3_	−2.36	−0.89	−0.75	−1.207
**8**	CF_3_	H	Fc	−2.96			−1.102
**9**	CF_3_	H	CH_3_	−3.21	−0.32	−0.32	−0.907
**10**	CF_3_	H	C_4_H_3_O	−3.07			−0.881
**11**	CF_3_	H	C_4_H_3_SC_4_H_2_S	−3.01			−0.852
**12**	CF_3_	H	Ph	−3.12	−0.27	−0.34	−0.838
**13**	CF_3_	H	C_4_H_3_S	−3.11	−0.27		−0.815
**14**	CF_3_	H	CF_3_	−4.14	−0.05	0.27	−0.473
**15**	Ph	H	H	−2.52		−0.63	
**16**	H	H	C(CH_3_)_3_	−2.50		−0.56	
**17**	CH_3_	NO_2_	CH_3_	−3.41		−0.08	
**18**	CH_3_	CN	CH_3_	−3.35		−0.19	
**19**	Ph	NO_2_	CH_3_	−3.33		0.21	
**20**	CH_3_	H	C(CH_3_)_3_	−2.33		−0.88	
**21**	H	H	C_10_H_7_	−2.50		−0.53	
**22**	CH_3_	H	C_10_H_7_	−2.35		−0.72	

[Cu^II^(β-diketonato)(dmeen)]^+^							

**23**	C(CH_3_)_3_	H	C(CH_3_)_3_	−6.07	−0.73		
**24**	CH_3_	H	CH_3_	−6.20	−0.64		
**25**	Ph	H	CH_3_	−6.08	−0.59		
**26**	Ph	H	Ph	−5.99	−0.58		
**27**	CF_3_	H	CH_3_	−6.63	−0.45		
**28**	CF_3_	H	Ph	−6.45	−0.38		
**29**	CF_3_	H	C_4_H_3_S	−6.42	−0.41		
**30**	CF_3_	H	CF_3_	−7.07	−0.16		

a *E*_pc_ obtained from cyclic voltammetry data, in DMSO as solvent, with a carbon fibre working electrode, from reference [8].

b *E*_pc_ obtained from cyclic voltammetry data, in acetonitrile as solvent, with a carbon fibre working electrode, from reference [7].

c *E*_pc_ obtained from cyclic voltammetry data, in acetonitrile as solvent, with a glassy carbon working electrode, from references [4], [5], [6].

d Fc = ferrocene = Fe(η^5^-C_5_H_5_)_2_; Ph = phenyl = C_5_H_6_.

## 2 Experimental Design, Materials and Methods

The optimized geometry of the specified molecules were obtained by DFT calculations, similar to the computations described in our previous publication [12]. The Gaussian 16 package [13] was used, together with the hybrid functional B3LYP [14,15], while applying the GTO (Gaussian type orbital) triple-ζ basis set 6–311G(d,p) for all the atoms. The optimization of the molecules was done in the gas phase. The Berny optimization algorithm [16] was used, requesting a convergence on energy of 1.0D-8 atomic unit. The input coordinates for the compounds were constructed using Chemcraft software [17]. The coordinates and multiplicity (2) were specified in the input files of the DFT calculations. Frequency calculations were done on all molecules to ensure true minimum energy (no imaginary frequency). The LUMO energies were obtained from the output files, searching for “Orbital energies and kinetic energies” from the bottom of the file and identifying the LUMO from the orbital energies and the provided occupations.

## Ethics Statement

This work does not require any ethical statement.

## CRediT Author Statement

**Marrigje M Conradie:** Conceptualization, Methodology, Writing - review & editing; **Ernst H.G. Langner:** Writing - review & editing; **Jeanet Conradie:** Conceptualization, Methodology, Writing - review & editing.

## Supplementary data files

Optimized coordinates (xyz) of the molecules.

## Declaration of Competing Interest

The authors declare no known competing financial interests or personal relationships which have or could be perceived to have influenced the work reported in this article.

## Appendix Supplementary materials

Image, application 1
