# Supplementary material for: DFT data to relate calculated LUMO energy with experimental reduction potentials of Cu(II)-β-diketonato complexes
Source: Data Brief. 2021 Aug 27;38:107331. doi: 10.1016/j.dib.2021.107331 (PMC8411210; doi:10.1016/j.dib.2021.107331)
Supplement: Supplementary file 1 [file mmc1.pdf]

DFT data to relate calculated LUMO energy with experimental reduction potentials of Cu(II)-β-diketonato complexes

Authors

Marrigje M. Conradie, Ernst H.G. Langner, Jeanet Conradie

Affiliations

Department of Chemistry, Faculty of Natural and Agricultural Sciences, PO Box 339, University of the Free State, Bloemfontein, 9300, South Africa.

Corresponding author(s)

Marrigje M Conradie (ConradieMM@ufs.ac.za)

Supporting information

Optimized Cartesian coordinates in angstrom, Å (1 Å = 10^{-10} m).

All compounds were optimized the hybrid functional B3LYP with the GTO (Gaussian type orbital) triple-ζ basis set 6-311G(d,p)

Table of Contents

Table of Contents 1
1. [Cu((C(CH$_3$)$_3$)COCHCOC(CH$_3$)$_3$)$_2$] 3
2. [Cu(FcCOCHCOCH$_3$)$_2$] 4
3. [Cu(FcCOCHCOFc)$_2$] 5
4. [Cu(FcCOCHCOPh)$_2$] 7
5. [Cu((CH$_3$)COCHCOCH$_3$)$_2$] 9
6. [Cu(PhCOCHCOPh)$_2$] 9
7. [Cu(PhCOCHCOCH$_3$)$_2$] 10
8. [Cu((CF$_3$)COCHCOFc)$_2$] 11
9. [Cu((CF$_3$)COCHCOCH$_3$)$_2$] 13
10. [Cu((CF$_3$)COCHCOC$_4$H$_3$O)$_2$] 13
11. [Cu((CF$_3$)COCHCOC$_4$H$_3$SC$_4$H$_2$S)$_2$] 14
12. [Cu((CF$_3$)COCHCOPh)$_2$] 15
13. [Cu((CF$_3$)COCHCOC$_4$H$_3$S)$_2$] 16
14. [Cu((CF$_3$)COCHCOCF$_3$)$_2$] 17
15. [Cu((C$_6$H$_5$)COCHCOH)$_2$] 18
16. [Cu(HCOCHCOC(CH$_3$)$_3$)$_2$] 18
17. [Cu((CH$_3$)COC(NO$_2$)COCH$_3$)$_2$] 19
18. [Cu((CH$_3$)COC(CN)COCH$_3$)$_2$] 20

19. [Cu((C$_6$H$_5$)COC(NO$_2$)COCH$_3$)$_2$] 21
20. [Cu((CH$_3$)COCHCOC(CH$_3$)$_3$)$_2$] 22
21. [Cu(HCOCHCO(C$_{10}$H$_7$))$_2$] 23
22. [Cu((CH$_3$)COCHCO(C$_{10}$H$_7$))$_2$] 24
23. [Cu((CF$_3$)COCHCOCF$_3$)(dmeen)]$^+$ 25
24. [Cu((CH$_3$)COCHCOCH$_3$)(dmeen)]$^+$ 26
25. [Cu(PhCOCHCOCH$_3$)(dmeen)]$^+$ 27
26. [Cu(PhCOCHCOPh)(dmeen)]$^+$ 28
27. [Cu((CF$_3$)COCHCOCH$_3$)(dmeen)]$^+$ 29
28. [Cu((CF$_3$)COCHCOPh)(dmeen)]$^+$ 29
29. [Cu((CF$_3$)COCHCOC$_4$H$_3$S)(dmeen)]$^+$ 30
30. [Cu((CF$_3$)COCHCOCF$_3$)(dmeen)]$^+$ 31

1. [Cu((C(CH$_3$)$_3$)COCHCOC(CH$_3$)$_3$)$_2$]

Cu	0.703443193	-0.093794173	-0.000079277
O	-0.638356807	-1.469169173	-0.000750277
O	-0.638149807	1.281588827	0.000148723
C	-2.569018807	-0.093641173	0.000052723
H	-3.643670807	-0.093579173	0.000236723
C	-2.674763807	2.479750827	-0.000307277
C	-1.902716807	-1.330430173	-0.000101277
C	-2.675147807	-2.667039173	0.000358723
C	-1.902559807	1.143032827	-0.000114277
O	2.045245193	1.281573827	0.000864723
O	2.045040193	-1.469178173	-0.000548277
C	3.975909193	-0.093947173	-0.000034277
H	5.050561193	-0.094013173	-0.000086277
C	4.081655193	-2.667336173	-0.000185277
C	3.309606193	1.142840827	0.000270723
C	4.082029193	2.479452827	0.000227723
C	3.309450193	-1.330621173	-0.000213277
C	-2.246031807	3.262583827	1.260807723
H	-2.547932807	2.737059827	2.171847723
H	-1.163681807	3.391838827	1.281704723
H	-2.719346807	4.248734827	1.267841723
C	-4.202166807	2.313282827	-0.000764277
H	-4.674844807	3.299447827	-0.001312277
H	-4.554355807	1.779586827	-0.887401277
H	-4.554972807	1.780296827	0.886056723
C	-2.245328807	3.262724827	-1.261073277
H	-1.162956807	3.391871827	-1.281414277
H	-2.546855807	2.737390827	-2.172345277
H	-2.718526807	4.248931827	-1.268152277
C	3.653620193	3.262110827	1.261557723
H	3.955633193	2.736406827	2.172454723
H	4.127040193	4.248208827	1.268646723
H	2.571286193	3.391487827	1.282656723
C	3.652492193	3.262745827	-1.260302277
H	4.125799193	4.248902827	-1.267245277
H	3.953825193	2.737584827	-2.171742277
H	2.570130193	3.392020827	-1.280445277
C	5.609420193	2.312772827	-0.000525277
H	5.961416193	1.779536827	-0.887513277
H	6.082202193	3.298891827	-0.000638277
H	5.962298193	1.779291827	0.885963723
C	5.609057193	-2.500862173	0.000714723
H	6.081737193	-3.487027173	0.000941723
H	5.962079193	-1.967479173	-0.885781277
H	5.961029193	-1.967562173	0.887675723
C	3.651904193	-3.450657173	1.260254723
H	2.569515193	-3.579695173	1.280348723
H	4.125006193	-4.436911173	1.267122723
H	3.953309193	-2.925628173	2.171743723

C	3.653246193	-3.449824173	-1.261629277
H	3.955343193	-2.924029173	-2.172449277
H	4.126599193	-4.435954173	-1.268832277
H	2.570906193	-3.579113173	-1.282819277
C	-2.245537807	-3.449966173	1.261091723
H	-2.718853807	-4.436116173	1.268356723
H	-1.163175807	-3.579252173	1.281196723
H	-2.546803807	-2.924535173	2.172398723
C	-2.246830807	-3.450067173	-1.260772277
H	-2.548895807	-2.924620173	-2.171799277
H	-1.164500807	-3.579463173	-1.281906277
H	-2.720267807	-4.436160173	-1.267546277
C	-4.202538807	-2.500346173	0.001161723
H	-4.554482807	-1.966863173	0.888020723
H	-4.555461807	-1.967108173	-0.885455277
H	-4.675325807	-3.486463173	0.001576723

2. [Cu(FcCOCHCOCH$_3$)$_2$]

Cu	0.000000000	0.000000000	0.000000000
Fe	-4.417368000	-0.205994000	-3.198692000
O	1.100694000	-0.710397000	-1.411422000
O	-1.608991000	-0.625551000	-0.842772000
C	1.805433000	-1.753501000	-3.395855000
H	2.390293000	-0.879825000	-3.695446000
H	1.426533000	-2.258102000	-4.284775000
H	2.482050000	-2.424399000	-2.859832000
C	0.702616000	-1.312905000	-2.460577000
C	-0.631469000	-1.583664000	-2.786916000
H	-0.833993000	-2.116209000	-3.704174000
C	-1.715799000	-1.223622000	-1.963686000
C	-3.095396000	-1.555990000	-2.370533000
C	-4.236042000	-1.389046000	-1.518272000
H	-4.189777000	-1.012836000	-0.509038000
C	-5.392293000	-1.773299000	-2.245264000
H	-6.408843000	-1.738476000	-1.883459000
C	-4.984244000	-2.172939000	-3.552495000
H	-5.636871000	-2.491087000	-4.351612000
C	-3.572390000	-2.038700000	-3.636094000
H	-2.973860000	-2.240170000	-4.510754000
C	-5.301792000	1.618049000	-2.773473000
H	-5.943040000	1.797931000	-1.923960000
C	-3.883972000	1.748946000	-2.792445000
H	-3.261088000	2.029161000	-1.956431000
C	-3.425153000	1.373517000	-4.089716000
H	-2.394902000	1.335918000	-4.409915000
C	-4.559754000	1.009619000	-4.870668000
H	-4.541577000	0.653558000	-5.889631000
C	-5.720392000	1.159476000	-4.056756000
H	-6.735135000	0.939156000	-4.352045000

Fe	4.417368000	0.205994000	3.198692000
O	-1.100694000	0.710397000	1.411422000
O	1.608991000	0.625551000	0.842772000
C	-1.805433000	1.753501000	3.395855000
H	-2.390293000	0.879825000	3.695446000
H	-1.426533000	2.258102000	4.284775000
H	-2.482050000	2.424399000	2.859832000
C	-0.702616000	1.312905000	2.460577000
C	0.631469000	1.583664000	2.786916000
H	0.833993000	2.116209000	3.704174000
C	1.715799000	1.223622000	1.963686000
C	3.095396000	1.555990000	2.370533000
C	4.236042000	1.389046000	1.518272000
H	4.189777000	1.012836000	0.509038000
C	5.392293000	1.773299000	2.245264000
H	6.408843000	1.738476000	1.883459000
C	4.984244000	2.172939000	3.552495000
H	5.636871000	2.491087000	4.351612000
C	3.572390000	2.038700000	3.636094000
H	2.973860000	2.240170000	4.510754000
C	5.301792000	-1.618049000	2.773473000
H	5.943040000	-1.797931000	1.923960000
C	3.883972000	-1.748946000	2.792445000
H	3.261088000	-2.029161000	1.956431000
C	3.425153000	-1.373517000	4.089716000
H	2.394902000	-1.335918000	4.409915000
C	4.559754000	-1.009619000	4.870668000
H	4.541577000	-0.653558000	5.889631000
C	5.720392000	-1.159476000	4.056756000
H	6.735135000	-0.939156000	4.352045000

3. [Cu(FcCOCHCOFc)$_2$]

C	3.391417000	-4.782620000	1.725286000
C	4.775869000	-4.989527000	1.454801000
C	5.423543000	-3.720450000	1.482381000
C	4.439157000	-2.730539000	1.766871000
C	3.182061000	-3.387193000	1.916330000
C	4.698489000	-4.071256000	-1.879548000
C	3.452481000	-4.746218000	-1.717611000
C	2.462678000	-3.778780000	-1.403330000
C	3.088080000	-2.489181000	-1.378185000
C	4.480437000	-2.683176000	-1.668931000
C	4.769671000	4.994718000	1.454536000
C	5.418500000	3.726254000	1.483314000
C	4.434793000	2.735595000	1.767591000
C	3.176979000	3.391183000	1.915718000
C	3.385201000	4.786684000	1.724064000
C	4.478770000	2.686262000	-1.668133000
C	4.695790000	4.074399000	-1.879388000

C	3.449078000	4.748386000	-1.718796000
C	2.459861000	3.780269000	-1.404687000
C	3.086375000	2.491228000	-1.378313000
C	2.372576000	1.237164000	-1.060527000
C	2.997223000	0.001015000	-1.293539000
C	2.373439000	-1.235574000	-1.060498000
Cu	0.000001000	0.000000000	-0.000010000
Fe	3.947253000	-3.734537000	0.027406000
Fe	3.943340000	3.738176000	0.027189000
O	1.188273000	1.384957000	-0.603675000
O	1.189234000	-1.384151000	-0.603663000
H	2.628616000	-5.546101000	1.745686000
H	5.246207000	-5.937464000	1.241343000
H	6.470440000	-3.538657000	1.291654000
H	4.607762000	-1.665976000	1.825490000
H	2.231579000	-2.905569000	2.089400000
H	5.649338000	-4.536424000	-2.092097000
H	3.296932000	-5.812133000	-1.789512000
H	1.419270000	-3.953071000	-1.196738000
H	5.239880000	-1.917486000	-1.696570000
H	5.239267000	5.942982000	1.240890000
H	6.465708000	3.545335000	1.293464000
H	4.604334000	1.671228000	1.827015000
H	2.226811000	2.908728000	2.088201000
H	2.621690000	5.549481000	1.743445000
H	5.238889000	1.921200000	-1.694608000
H	5.646401000	4.540279000	-2.091448000
H	3.292647000	5.814115000	-1.791532000
H	1.416153000	3.953817000	-1.198981000
H	3.984488000	0.001372000	-1.728732000
C	-3.391422000	4.782644000	-1.725276000
C	-4.775871000	4.989554000	-1.454780000
C	-5.423550000	3.720480000	-1.482365000
C	-4.439170000	2.730568000	-1.766869000
C	-3.182073000	3.387218000	-1.916332000
C	-4.698473000	4.071258000	1.879561000
C	-3.452464000	4.746216000	1.717621000
C	-2.462666000	3.778777000	1.403327000
C	-3.088074000	2.489180000	1.378176000
C	-4.480428000	2.683179000	1.668933000
C	-4.769681000	-4.994744000	-1.454515000
C	-5.418515000	-3.726282000	-1.483296000
C	-4.434813000	-2.735622000	-1.767588000
C	-3.176999000	-3.391207000	-1.915721000
C	-3.385215000	-4.786707000	-1.724056000
C	-4.478761000	-2.686265000	1.668136000
C	-4.695776000	-4.074401000	1.879401000
C	-3.449063000	-4.748385000	1.718804000
C	-2.459852000	-3.780268000	1.404680000
C	-3.086369000	-2.491228000	1.378301000
C	-2.372578000	-1.237165000	1.060499000

C	-2.997221000	-0.001016000	1.293519000
C	-2.373439000	1.235573000	1.060472000
Fe	-3.947251000	3.734550000	-0.027400000
Fe	-3.943343000	-3.738189000	-0.027183000
O	-1.188274000	-1.384958000	0.603651000
O	-1.189233000	1.384150000	0.603641000
H	-2.628618000	5.546123000	-1.745675000
H	-5.246204000	5.937492000	-1.241312000
H	-6.470447000	3.538689000	-1.291633000
H	-4.607779000	1.666006000	-1.825496000
H	-2.231594000	2.905592000	-2.089412000
H	-5.649319000	4.536428000	2.092119000
H	-3.296910000	5.812130000	1.789529000
H	-1.419259000	3.953066000	1.196729000
H	-5.239873000	1.917491000	1.696571000
H	-5.239273000	-5.943008000	-1.240858000
H	-6.465722000	-3.545365000	-1.293438000
H	-4.604358000	-1.671256000	-1.827018000
H	-2.226833000	-2.908750000	-2.088215000
H	-2.621701000	-5.549502000	-1.743438000
H	-5.238882000	-1.921205000	1.694612000
H	-5.646383000	-4.540282000	2.091473000
H	-3.292628000	-5.814113000	1.791546000
H	-1.416145000	-3.953814000	1.198967000
H	-3.984481000	-0.001373000	1.728724000

4. [Cu(FcCOCHCOPh)$_2$]

Cu	0.000000000	0.000000000	0.000000000
Fe	-4.470026000	-0.152710000	-3.161719000
O	1.068500000	-0.695032000	-1.437681000
O	-1.629038000	-0.539781000	-0.861759000
C	0.668296000	-1.304930000	-2.485246000
C	-0.675530000	-1.575721000	-2.782635000
H	-0.906985000	-2.158020000	-3.659724000
C	-1.746545000	-1.173052000	-1.963583000
C	-3.130933000	-1.502427000	-2.358101000
C	-4.264080000	-1.349629000	-1.493164000
H	-4.211421000	-0.989015000	-0.478711000
C	-5.425368000	-1.734844000	-2.211702000
H	-6.438204000	-1.710638000	-1.838789000
C	-5.028416000	-2.119828000	-3.526524000
H	-5.687400000	-2.433805000	-4.322015000
C	-3.618432000	-1.975938000	-3.623144000
H	-3.027833000	-2.164074000	-4.506058000
C	-5.312954000	1.677892000	-2.682975000
H	-5.899832000	1.863138000	-1.796155000
C	-3.898225000	1.798766000	-2.789351000
H	-3.222765000	2.076283000	-1.994481000
C	-3.522887000	1.420987000	-4.112360000

H	-2.514808000	1.379032000	-4.496241000
C	-4.706269000	1.066051000	-4.821741000
H	-4.753592000	0.711331000	-5.840259000
C	-5.813274000	1.222696000	-3.938005000
H	-6.845784000	1.009656000	-4.170189000
Fe	4.470026000	0.152710000	3.161719000
O	-1.068500000	0.695032000	1.437681000
O	1.629038000	0.539781000	0.861759000
C	-0.668296000	1.304930000	2.485246000
C	0.675530000	1.575721000	2.782635000
H	0.906985000	2.158020000	3.659724000
C	1.746545000	1.173052000	1.963583000
C	3.130933000	1.502427000	2.358101000
C	4.264080000	1.349629000	1.493164000
H	4.211421000	0.989015000	0.478711000
C	5.425368000	1.734844000	2.211702000
H	6.438204000	1.710638000	1.838789000
C	5.028416000	2.119828000	3.526524000
H	5.687400000	2.433805000	4.322015000
C	3.618432000	1.975938000	3.623144000
H	3.027833000	2.164074000	4.506058000
C	5.312954000	-1.677892000	2.682975000
H	5.899832000	-1.863138000	1.796155000
C	3.898225000	-1.798766000	2.789351000
H	3.222765000	-2.076283000	1.994481000
C	3.522887000	-1.420987000	4.112360000
H	2.514808000	-1.379032000	4.496241000
C	4.706269000	-1.066051000	4.821741000
H	4.753592000	-0.711331000	5.840259000
C	5.813274000	-1.222696000	3.938005000
H	6.845784000	-1.009656000	4.170189000
C	1.755964000	-1.758644000	-3.408647000
C	3.067569000	-1.809421000	-2.917342000
C	1.523236000	-2.117105000	-4.743258000
C	4.113835000	-2.227813000	-3.731260000
C	2.572362000	-2.527241000	-5.559706000
C	3.869972000	-2.589511000	-5.055259000
H	3.241485000	-1.513278000	-1.891247000
H	0.525539000	-2.054474000	-5.158939000
H	5.122009000	-2.269980000	-3.334313000
H	2.377320000	-2.793789000	-6.592366000
H	4.686084000	-2.912360000	-5.691986000
C	-1.755964000	1.758644000	3.408647000
C	-3.067569000	1.809421000	2.917342000
C	-1.523236000	2.117105000	4.743258000
C	-4.113835000	2.227813000	3.731260000
C	-2.572362000	2.527241000	5.559706000
C	-3.869972000	2.589511000	5.055259000
H	-3.241485000	1.513278000	1.891247000
H	-0.525539000	2.054474000	5.158939000
H	-5.122009000	2.269980000	3.334313000

H	-2.377320000	2.793789000	6.592366000
H	-4.686084000	2.912360000	5.691986000

5. [Cu((CH$_3$)COCHCOCH$_3$)$_2$]

Cu	0.000000000	0.000000000	3.267954949
O	1.079456098	0.868330923	4.605824700
O	-1.067539692	-0.881989311	4.605701167
C	0.000000000	0.000000000	6.535909898
H	-0.002811390	0.004266619	7.616594745
C	-1.951182412	-1.592651266	6.662641769
H	-1.855175906	-2.646320586	6.387900620
H	-2.962632935	-1.281191638	6.388648268
H	-1.817995414	-1.484784081	7.739176784
C	0.958748634	0.777075758	5.867176203
C	1.952114586	1.590501491	6.664103442
H	1.851770249	2.644051184	6.390125313
H	2.965247277	1.283669853	6.391439005
H	1.816888169	1.481198495	7.740168985
C	-0.953958058	-0.782721134	5.867258779
O	-1.079456098	-0.868330923	1.930085198
O	1.067539693	0.881989311	1.930208731
C	0.000000000	0.000000000	0.000000000
H	0.002811391	-0.004266619	-1.080684846
C	1.951182412	1.592651266	-0.126731870
H	1.855175906	2.646320586	0.148009279
H	2.962632935	1.281191638	0.147261630
H	1.817995415	1.484784081	-1.203266886
C	-0.958748634	-0.777075758	0.668733696
C	-1.952114586	-1.590501491	-0.128193544
H	-1.851770249	-2.644051184	0.145784586
H	-2.965247277	-1.283669853	0.144470893
H	-1.816888169	-1.481198495	-1.204259087
C	0.953958058	0.782721134	0.668651120

6. [Cu(PhCOCHCOPh)$_2$]

Cu	-0.072359267	-0.054822799	0.600410597
O	-0.123605128	-1.433836515	1.934965664
O	-0.160826367	1.322093147	1.935341026
C	-0.072359237	-0.054822837	3.867696398
H	0.039677984	-0.054822799	4.939285238
C	-0.091919488	-1.292240921	3.201552346
C	-0.121346946	1.182022669	3.201976445
O	-0.021113407	1.324190916	-0.734144470
O	0.016107832	-1.431738746	-0.734519833
C	-0.072359298	-0.054822762	-2.666875205
H	-0.184396519	-0.054822799	-3.738464045
C	-0.052799047	1.182595322	-2.000731152

C	-0.023371589	-1.291668268	-2.001155252
C	-0.044880165	-2.567974251	3.980988956
C	-0.428690691	-2.650043080	5.326040289
C	0.386551558	-3.731308664	3.329141423
C	-0.371275858	-3.863076384	6.004848035
C	0.454484835	-4.940131117	4.011877206
C	0.076317855	-5.010077762	5.351974594
H	-0.799835979	-1.772517356	5.840554374
H	0.665595011	-3.662821088	2.285772725
H	-0.680859134	-3.914042019	7.042615480
H	0.800671659	-5.830228969	3.498540000
H	0.124610757	-5.954033701	5.883331257
C	-0.044470142	-2.568833939	-2.779473289
C	-0.431443183	-3.743440269	-2.119848286
C	0.318863742	-2.641641408	-4.130782876
C	-0.475668952	-4.954709328	-2.800152151
C	0.284194941	-3.856564437	-4.807688496
C	-0.118885876	-5.015287630	-4.146541018
H	-0.695905335	-3.681144452	-1.072300420
H	0.655936438	-1.754891808	-4.652700487
H	-0.787303903	-5.853691084	-2.280241403
H	0.577075551	-3.899765978	-5.850645336
H	-0.149345234	-5.960668512	-4.676677401
C	-0.099838370	2.458328652	-2.780167762
C	0.283972156	2.540397481	-4.125219096
C	-0.531270093	3.621663065	-2.128320230
C	0.226557323	3.753430785	-4.804026841
C	-0.599203370	4.830485518	-2.811056012
C	-0.221036390	4.900432163	-4.151153400
H	0.655117444	1.662871757	-4.639733181
H	-0.810313546	3.553175489	-1.084951532
H	0.536140599	3.804396420	-5.841794286
H	-0.945390194	5.720583370	-2.297718807
H	-0.269329292	5.844388102	-4.682510064
C	-0.100248393	2.459188340	3.980294483
C	0.286724648	3.633794671	3.320669479
C	-0.463582277	2.531995809	5.331604069
C	0.330950417	4.845063729	4.000973344
C	-0.428913476	3.746918838	6.008509689
C	-0.025832659	4.905642031	5.347362212
H	0.551186800	3.571498853	2.273121614
H	-0.800654973	1.645246209	5.853521680
H	0.642585368	5.744045485	3.481062596
H	-0.721794086	3.790120379	7.051466529
H	0.004626699	5.851022913	5.877498595

7. [Cu(PhCOCHCOCH$_3$)$_2$]

Cu	0.000000000	0.000000000	0.000000000
O	0.975880000	1.559296000	0.561968000

O	0.871553000	-0.004622000	-1.710230000
C	1.898245000	2.153767000	-0.081344000
C	2.340586000	1.821954000	-1.370215000
H	3.151044000	2.401398000	-1.783532000
C	1.793715000	0.769369000	-2.126230000
C	2.292295000	0.475393000	-3.506445000
C	3.087521000	1.366425000	-4.239827000
H	3.357051000	2.329143000	-3.824732000
C	3.517870000	1.039531000	-5.521734000
H	4.126462000	1.742225000	-6.079650000
C	3.164105000	-0.183020000	-6.089622000
H	3.502909000	-0.436858000	-7.087888000
C	2.368028000	-1.073836000	-5.371240000
H	2.085713000	-2.024673000	-5.809170000
C	1.931112000	-0.745406000	-4.093447000
H	1.302242000	-1.419012000	-3.526185000
C	2.536686000	3.312186000	0.649901000
H	1.765121000	4.045924000	0.897798000
H	3.321498000	3.795666000	0.068174000
H	2.953351000	2.952659000	1.594436000
O	-0.975880000	-1.559296000	-0.561968000
C	-1.898245000	-2.153767000	0.081344000
C	-2.340586000	-1.821954000	1.370215000
H	-3.151044000	-2.401398000	1.783532000
C	-1.793715000	-0.769369000	2.126230000
O	-0.871553000	0.004622000	1.710230000
C	-2.292295000	-0.475393000	3.506445000
C	-3.087521000	-1.366425000	4.239827000
H	-3.357051000	-2.329143000	3.824732000
C	-3.517870000	-1.039531000	5.521734000
H	-4.126462000	-1.742225000	6.079650000
C	-3.164105000	0.183020000	6.089622000
H	-3.502909000	0.436858000	7.087888000
C	-2.368028000	1.073836000	5.371240000
H	-2.085713000	2.024673000	5.809170000
C	-1.931112000	0.745406000	4.093447000
H	-1.302242000	1.419012000	3.526185000
C	-2.536686000	-3.312186000	-0.649901000
H	-1.765121000	-4.045924000	-0.897798000
H	-3.321498000	-3.795666000	-0.068174000
H	-2.953351000	-2.952659000	-1.594436000

8. $[Cu((CF_3)COCHCOFc)_2]$

Cu	0.000000000	0.000000000	0.000000000
Fe	-4.417041000	-0.204942000	-3.212169000
O	1.094293000	-0.695767000	-1.422362000
O	-1.606772000	-0.643568000	-0.834228000
C	1.783437000	-1.692077000	-3.419030000
C	0.654728000	-1.274385000	-2.463834000

C	-0.654088000	-1.555159000	-2.809526000
H	-0.847168000	-2.065342000	-3.738712000
C	-1.741266000	-1.217689000	-1.959911000
C	-3.114445000	-1.551512000	-2.354688000
C	-4.258139000	-1.357070000	-1.508776000
H	-4.215833000	-0.966923000	-0.504649000
C	-5.410876000	-1.749694000	-2.233656000
H	-6.428681000	-1.701568000	-1.877308000
C	-5.000598000	-2.176386000	-3.531925000
H	-5.653046000	-2.505327000	-4.326577000
C	-3.589731000	-2.052876000	-3.615986000
H	-2.989300000	-2.281178000	-4.482582000
C	-5.310664000	1.624831000	-2.827785000
H	-5.953900000	1.819635000	-1.983152000
C	-3.894112000	1.761751000	-2.846539000
H	-3.277032000	2.071106000	-2.016574000
C	-3.430978000	1.362018000	-4.135731000
H	-2.400868000	1.327865000	-4.456990000
C	-4.562121000	0.977243000	-4.910255000
H	-4.540105000	0.600837000	-5.921753000
C	-5.724536000	1.136737000	-4.101411000
H	-6.737565000	0.904995000	-4.393478000
Fe	4.417041000	0.204942000	3.212169000
O	-1.094293000	0.695767000	1.422362000
O	1.606772000	0.643568000	0.834228000
C	-1.783437000	1.692077000	3.419030000
C	-0.654728000	1.274385000	2.463834000
C	0.654088000	1.555159000	2.809526000
H	0.847168000	2.065342000	3.738712000
C	1.741266000	1.217689000	1.959911000
C	3.114445000	1.551512000	2.354688000
C	4.258139000	1.357070000	1.508776000
H	4.215833000	0.966923000	0.504649000
C	5.410876000	1.749694000	2.233656000
H	6.428681000	1.701568000	1.877308000
C	5.000598000	2.176386000	3.531925000
H	5.653046000	2.505327000	4.326577000
C	3.589731000	2.052876000	3.615986000
H	2.989300000	2.281178000	4.482582000
C	5.310664000	-1.624831000	2.827785000
H	5.953900000	-1.819635000	1.983152000
C	3.894112000	-1.761751000	2.846539000
H	3.277032000	-2.071106000	2.016574000
C	3.430978000	-1.362018000	4.135731000
H	2.400868000	-1.327865000	4.456990000
C	4.562121000	-0.977243000	4.910255000
H	4.540105000	-0.600837000	5.921753000
C	5.724536000	-1.136737000	4.101411000
H	6.737565000	-0.904995000	4.393478000
F	1.330431000	-2.310400000	-4.530053000
F	2.487449000	-0.617092000	-3.815878000

F	2.632669000	-2.535737000	-2.806301000
F	-1.330431000	2.310400000	4.530053000
F	-2.487449000	0.617092000	3.815878000
F	-2.632669000	2.535737000	2.806301000

9. [Cu((CF$_3$)COCHCOCH$_3$)$_2$]

Cu	-0.210105697	-0.183322980	0.321311614
O	-1.843031697	0.835828020	0.259229614
O	0.522973303	0.793169020	-1.168091386
C	-1.309283697	2.277336020	-1.547192386
H	-1.654789697	3.091160020	-2.164499386
C	0.811672303	2.317204020	-2.915688386
C	-2.148432697	1.782289020	-0.523009386
C	-3.510271697	2.392056020	-0.310043386
H	-3.573100697	2.773126020	0.712514614
H	-4.268697697	1.611099020	-0.407553386
H	-3.722378697	3.196922020	-1.012916386
C	-0.051395697	1.739846020	-1.780423386
O	1.422820303	-1.202473980	0.383393614
O	-0.943184697	-1.159814980	1.810714614
C	0.889072303	-2.643981980	2.189815614
H	1.234578303	-3.457805980	2.807122614
C	-1.231883697	-2.683849980	3.558311614
C	1.728221303	-2.148934980	1.165632614
C	3.090060303	-2.758701980	0.952666614
H	3.152889303	-3.139771980	-0.069891386
H	3.848486303	-1.977744980	1.050176614
H	3.302167303	-3.563567980	1.655539614
C	-0.368815697	-2.106491980	2.423046614
F	-2.400202697	-3.136944980	3.074762614
F	-1.500753697	-1.734901980	4.470897614
F	-0.632664697	-3.707379980	4.200098614
F	0.212453303	3.340734020	-3.557475386
F	1.080542303	1.368256020	-3.828274386
F	1.979991303	2.770299020	-2.432139386

10. [Cu((CF$_3$)COCHCOC$_4$H$_3$O)$_2$]

Cu	0.371081152	0.519554097	0.005349933
O	-1.310765848	1.451339097	0.005019933
O	-0.505188848	-1.195248903	0.004972933
C	-2.770777848	-0.436310903	0.006832933
H	-3.800022848	-0.755369903	0.007837933
C	-2.133459848	-2.872645903	0.006754933
C	-2.479199848	0.949164097	0.006015933
C	-1.759781848	-1.381600903	0.006143933
O	2.053036152	-0.412083903	0.005726933
O	1.247410152	2.234337097	0.005827933

C	3.513037152	1.475549097	0.006587933
H	4.542264152	1.794669097	0.006991933
C	3.221481152	0.090078097	0.006243933
C	2.501995152	2.420782097	0.006290933
F	-3.466241848	-3.077731903	0.008306933
F	-1.628342848	-3.483514903	1.092705933
F	-1.630834848	-3.483773903	-1.080222067
C	-3.555797848	3.283879097	0.005830933
C	-4.915757848	3.698990097	0.006606933
C	-5.659795848	2.555432097	0.007587933
O	-4.862595848	1.462526097	0.007454933
C	-3.568048848	1.914242097	0.006386933
H	-2.668049848	3.894703097	0.004980933
H	-5.294920848	4.708167097	0.006461933
H	-6.719752848	2.363149097	0.008404933
C	2.875464152	3.911871097	0.006764933
F	4.208207152	4.117179097	0.007156933
F	2.371164152	4.522460097	1.093275933
F	2.371762152	4.523126097	-1.079635067
C	4.298168152	-2.244624903	0.006220933
C	5.658169152	-2.659659903	0.006769933
C	6.402131152	-1.516069903	0.007192933
O	5.604877152	-0.423195903	0.007100933
C	4.310315152	-0.875009903	0.006517933
H	3.410501152	-2.855567903	0.005754933
H	6.037331152	-3.668836903	0.006791933
H	7.462077152	-1.323712903	0.007624933

11. $[Cu((CF_3)COCHCOC_4H_3SC_4H_2S)_2]$

Cu	-0.961813601	-0.000884820	0.004779886
O	-0.546357601	-1.878218820	-0.010049114
O	-2.851861601	-0.358466820	-0.012801114
C	-2.777984601	-2.736389820	-0.049743114
H	-3.350619601	-3.648773820	-0.069559114
C	-1.397624601	-2.819409820	-0.034122114
C	-0.736485601	-4.206622820	-0.045506114
C	-3.446812601	-1.484479820	-0.037864114
O	-1.377380601	1.876397180	0.018190886
O	0.928209399	0.356548180	0.023267886
C	0.854372399	2.734630180	0.049598886
C	-0.526010601	2.817636180	0.036767886
C	-1.187088601	4.204901180	0.044583886
C	1.523189399	1.482681180	0.041977886
C	3.913232399	2.446160180	0.083058886
C	2.978067399	1.431393180	0.053469886
S	3.753991399	-0.134343820	0.019394886
C	5.334633399	0.598880180	0.069963886
C	5.240414399	1.980099180	0.094004886
H	6.104716399	2.629148180	0.141008886

F	-0.288786601	5.213076180	0.065394886
F	-1.951320601	4.369406180	-1.048892114
F	-1.975662601	4.345999180	1.123579886
F	0.054913399	-4.343563820	-1.123001114
F	0.024897399	-4.375304820	1.049296886
F	-1.634696601	-5.214723820	-0.072548114
C	-7.163959601	-1.981655820	-0.098206114
C	-7.258202601	-0.600534820	-0.069421114
S	-5.677630601	0.132495180	-0.013388114
C	-4.901678601	-1.433126820	-0.051714114
C	-5.836799601	-2.447767820	-0.086544114
H	-8.028183601	-2.630518820	-0.149095114
H	-5.575710601	-3.496784820	-0.115169114
H	3.652166399	3.495264180	0.108483886
H	1.427058399	3.647080180	0.064259886
C	6.666730399	-1.542591820	0.425669886
C	6.530071399	-0.216471820	0.081042886
S	8.082297399	0.451014180	-0.387630114
C	8.875223399	-1.058986820	-0.095971114
C	7.999387399	-2.019725820	0.323784886
H	5.836118399	-2.149377820	0.761659886
H	8.296147399	-3.032661820	0.559953886
C	-9.922486601	2.018916180	-0.319100114
C	-10.799020601	1.056840180	0.096130886
S	-10.006609601	-0.454137820	0.384051886
C	-8.453596601	0.214878180	-0.079824114
C	-8.589686601	1.542101180	-0.420410114
H	-10.218839601	3.032635180	-0.552408114
H	-11.862800601	1.140365180	0.256270886
H	-7.758522601	2.149938180	-0.753127114
H	9.938745399	-1.143042820	-0.257545114

12. [Cu((CF$_3$)COCHCOPh)$_2$]

Cu	0.008189104	0.492713091	0.001096747
O	1.848975104	-0.052612909	0.001754747
O	0.503751104	2.349929091	0.000447747
C	2.881635104	2.072863091	-0.000672253
H	3.802700104	2.629151091	-0.002122253
C	2.902814104	0.654908091	0.000909747
C	1.694387104	2.786069091	-0.000757253
O	-1.832612896	1.038033091	0.001541747
O	-0.487380896	-1.364495909	0.000207747
C	-2.865259896	-1.087455909	-0.000541253
H	-3.786317896	-1.643755909	-0.001872253
C	-2.886442896	0.330495091	0.000908747
C	-1.678001896	-1.800648909	-0.000802253
C	4.188785104	-0.098183909	0.001339747
C	5.442867104	0.529959091	0.004515747
C	4.133506104	-1.500445909	-0.001409253

C	6.609630104	-0.226043909	0.004813747
C	5.300807104	-2.252866909	-0.001236253
C	6.542334104	-1.618111909	0.001857747
H	5.521640104	1.609035091	0.007107747
H	3.162338104	-1.977396909	-0.003662253
H	7.572199104	0.272070091	0.007398747
H	5.243364104	-3.335215909	-0.003480253
H	7.453896104	-2.205093909	0.002036747
C	-4.172424896	1.083577091	0.001340747
C	-5.426501896	0.455429091	0.005157747
C	-4.117158896	2.485839091	-0.002062253
C	-6.593270896	1.211424091	0.005447747
C	-5.284463896	3.238252091	-0.001921253
C	-6.525985896	2.603491091	0.001819747
H	-5.505270896	-0.623644909	0.008284747
H	-3.145995896	2.962795091	-0.004781253
H	-7.555833896	0.713303091	0.008561747
H	-5.227028896	4.320600091	-0.004689253
H	-7.437552896	3.190465091	0.001986747
C	1.751219104	4.322993091	-0.002369253
C	-1.734841896	-3.337572909	-0.002538253
F	-1.115580896	-3.829396909	-1.089227253
F	-1.116509896	-3.831837909	1.083601747
F	-2.996501896	-3.816686909	-0.003590253
F	1.131306104	4.814954091	-1.088643253
F	3.012848104	4.802148091	-0.004158253
F	1.133491104	4.817099091	1.084177747

13. [Cu((CF$_3$)COCHCOC$_4$H$_3$S)$_2$]

Cu	0.338625731	0.392191803	0.000161949
O	-0.128328269	2.257392803	0.000029949
O	-1.512762269	-0.131333197	-0.000055051
C	-2.508252269	2.027421803	0.000133949
H	-3.428256269	2.587825803	0.000213949
C	-1.309831269	2.718961803	0.000108949
C	-1.339543269	4.256051803	0.000146949
C	-2.546916269	0.609678803	0.000017949
O	0.805575731	-1.473009197	0.000308949
O	2.190011731	0.915722803	0.000346949
C	3.185499731	-1.243034197	-0.000194051
C	1.987078731	-1.934576197	-0.000005051
C	2.016793731	-3.471666197	-0.000114051
C	3.224163731	0.174707803	0.000054949
C	5.794495731	0.373791803	-0.000246051
C	4.507840731	0.871193803	0.000000949
S	4.511478731	2.617844803	0.000185949
C	6.232726731	2.645520803	0.000169949
C	6.780339731	1.387734803	-0.000151051
H	6.752000731	3.592133803	0.000326949

H	7.845136731	1.198211803	-0.000272051
F	3.271747731	-3.970037197	-0.000582051
F	1.391465731	-3.954809197	1.086516949
F	1.390696731	-3.954697197	-1.086340051
F	-0.713636269	4.739128803	1.086462949
F	-0.714024269	4.739147803	-1.086395051
F	-2.594496269	4.754424803	0.000372949
C	-6.103090269	-0.603351197	-0.000023051
C	-5.555477269	-1.861136197	0.000213949
S	-3.834229269	-1.833459197	-0.000450051
C	-3.830592269	-0.086808197	-0.000089051
C	-5.117247269	0.410593803	-0.000195051
H	-7.167887269	-0.413828197	0.000083949
H	-6.074750269	-2.807750197	0.000461949
H	-5.347640269	1.467046803	-0.000216051
H	6.024887731	-0.682662197	-0.000437051
H	4.105503731	-1.803438197	-0.000479051

14. [Cu((CF$_3$)COCHCOCF$_3$)$_2$]

Cu	0.090560779	-0.203001944	0.153852792
O	-1.544249221	0.816191056	0.093440792
O	0.816185779	0.789487056	-1.329899208
C	-1.015381221	2.263260056	-1.726008208
H	-1.376522221	3.071912056	-2.338667208
C	1.126031779	2.311749056	-3.082908208
C	-1.818445221	1.751400056	-0.704511208
C	-3.211229221	2.365613056	-0.463439208
C	0.254978779	1.729663056	-1.952791208
O	1.725370779	-1.222194944	0.214264792
O	-0.635064221	-1.195490944	1.637604792
C	1.196502779	-2.669263944	2.033713792
H	1.557643779	-3.477915944	2.646372792
C	-0.944910221	-2.717752944	3.390613792
C	1.999566779	-2.157403944	1.012216792
C	3.392350779	-2.771616944	0.771144792
C	-0.073857221	-2.135666944	2.260496792
F	-3.516340221	3.307137056	-1.375081208
F	-4.153563221	1.415709056	-0.517891208
F	-3.254727221	2.936104056	0.749940792
F	2.279945779	2.774449056	-2.582349208
F	1.407158779	1.360142056	-3.984274208
F	0.516257779	3.324694056	-3.725112208
F	4.334684779	-1.821712944	0.825596792
F	3.435848779	-3.342107944	-0.442235208
F	3.697461779	-3.713140944	1.682786792
F	-0.335136221	-3.730697944	4.032817792
F	-2.098824221	-3.180452944	2.890054792
F	-1.226037221	-1.766145944	4.291979792

15. [Cu((C$_6$H$_5$)COCHCOH)$_2$]

Cu	0.000000000	0.000000000	0.000000000
O	0.952062000	1.575798000	0.563826000
O	0.886444000	-0.009353000	-1.706242000
C	1.844960000	2.167308000	-0.113550000
C	2.301886000	1.856526000	-1.392958000
H	3.088782000	2.473881000	-1.798334000
C	1.787653000	0.777393000	-2.140548000
C	2.289416000	0.477166000	-3.516052000
C	3.128644000	1.343442000	-4.230291000
H	3.435287000	2.288830000	-3.801776000
C	3.563064000	1.011734000	-5.509277000
H	4.207698000	1.693997000	-6.051675000
C	3.168632000	-0.190584000	-6.093442000
H	3.510246000	-0.448169000	-7.089765000
C	2.330041000	-1.057142000	-5.393889000
H	2.017215000	-1.992212000	-5.844634000
C	1.890662000	-0.724860000	-4.118181000
H	1.232386000	-1.381084000	-3.564105000
H	2.306968000	3.032228000	0.381548000
O	-0.952062000	-1.575798000	-0.563826000
C	-1.844960000	-2.167308000	0.113550000
C	-2.301886000	-1.856526000	1.392958000
H	-3.088782000	-2.473881000	1.798334000
C	-1.787653000	-0.777393000	2.140548000
O	-0.886444000	0.009353000	1.706242000
C	-2.289416000	-0.477166000	3.516052000
C	-3.128644000	-1.343442000	4.230291000
H	-3.435287000	-2.288830000	3.801776000
C	-3.563064000	-1.011734000	5.509277000
H	-4.207698000	-1.693997000	6.051675000
C	-3.168632000	0.190584000	6.093442000
H	-3.510246000	0.448169000	7.089765000
C	-2.330041000	1.057142000	5.393889000
H	-2.017215000	1.992212000	5.844634000
C	-1.890662000	0.724860000	4.118181000
H	-1.232386000	1.381084000	3.564105000
H	-2.306968000	-3.032228000	-0.381548000

16. [Cu(HCOCHCOC(CH$_3$)$_3$)$_2$]

Cu	0.000002000	0.000000000	-0.000229000
O	-1.326930000	-1.397550000	-0.000884000
O	-1.349274000	1.371734000	-0.000331000
C	-3.270674000	0.000864000	-0.000754000
H	-4.348417000	-0.043890000	-0.000824000
C	-3.380198000	2.580546000	-0.000311000
C	-2.578767000	-1.212033000	-0.000947000
H	-3.184582000	-2.128868000	-0.001170000

C	-2.613709000	1.244644000	-0.000514000
O	1.326934000	1.397549000	0.000481000
O	1.349279000	-1.371735000	-0.000173000
C	3.270679000	-0.000864000	0.000409000
H	4.348422000	0.043890000	0.000576000
C	3.380204000	-2.580546000	-0.000064000
C	2.578772000	1.212033000	0.000599000
H	3.184586000	2.128868000	0.000891000
C	2.613714000	-1.244644000	0.000043000
C	-2.947273000	3.361403000	1.260845000
H	-3.249650000	2.836776000	2.172185000
H	-1.864401000	3.487226000	1.279524000
H	-3.417875000	4.348710000	1.268184000
C	-4.907413000	2.412450000	-0.000345000
H	-5.380811000	3.398090000	-0.000158000
H	-5.259119000	1.878704000	-0.887205000
H	-5.259125000	1.878371000	0.886312000
C	-2.947259000	3.361757000	-1.261234000
H	-1.864389000	3.487614000	-1.279852000
H	-3.249600000	2.837378000	-2.172728000
H	-3.417887000	4.349053000	-1.268305000
C	4.907419000	-2.412448000	0.000283000
H	5.380818000	-3.398087000	0.000142000
H	5.259304000	-1.878322000	-0.886277000
H	5.258948000	-1.878746000	0.887240000
C	2.947014000	-3.361819000	1.260735000
H	1.864138000	-3.487658000	1.279142000
H	3.417623000	-4.349124000	1.267844000
H	3.249189000	-2.837495000	2.172316000
C	2.947532000	-3.361339000	-1.261344000
H	3.250084000	-2.836662000	-2.172597000
H	3.418145000	-4.348642000	-1.268641000
H	1.864665000	-3.487170000	-1.280240000

17. [Cu((CH$_3$)COC(NO$_2$)COCH$_3$)$_2$]

Cu	0.000508000	0.000814000	0.000793000
O	-1.610611000	1.028564000	-0.074209000
O	0.702557000	1.001792000	-1.469839000
C	-1.102009000	2.465950000	-1.873871000
C	1.093201000	2.490584000	-3.225892000
H	0.782096000	2.094187000	-4.194464000
H	2.107484000	2.161364000	-3.005677000
H	1.048688000	3.576659000	-3.302172000
C	-1.948340000	1.978943000	-0.836672000
C	-3.333254000	2.520629000	-0.583210000
H	-3.263130000	3.455286000	-0.023259000
H	-3.870103000	1.780494000	0.008105000
H	-3.873750000	2.745074000	-1.502363000
C	0.195224000	1.943202000	-2.144384000

O	1.603915000	-1.039785000	0.063378000
O	-0.693904000	-0.987328000	1.483715000
C	1.101735000	-2.466397000	1.873388000
C	-1.065752000	-2.445420000	3.269364000
H	-1.817492000	-1.682714000	3.466439000
H	-1.555946000	-3.372144000	2.964589000
H	-0.500530000	-2.671617000	4.173115000
C	1.948120000	-1.979422000	0.836195000
C	3.306366000	-2.565278000	0.540132000
H	3.582596000	-2.256470000	-0.466839000
H	4.035197000	-2.177591000	1.254652000
H	3.326448000	-3.651119000	0.629136000
C	-0.194467000	-1.941888000	2.145511000
N	-1.591231000	3.561371000	-2.705576000
O	-1.350467000	3.526708000	-3.909726000
O	-2.220190000	4.465534000	-2.161486000
N	1.589885000	-3.563158000	2.703958000
O	0.795594000	-4.442682000	3.027434000
O	2.771560000	-3.554140000	3.039733000

18. [Cu((CH$_3$)COC(CN)COCH$_3$)$_2$]

Cu	0.000000000	0.000000000	0.000000000
O	-1.618495000	1.025147000	-0.067204000
O	0.708178000	0.990810000	-1.480350000
C	-1.105110000	2.474453000	-1.879513000
C	1.053278000	2.489433000	-3.243624000
H	1.301188000	1.672421000	-3.925873000
H	1.992887000	2.838452000	-2.807793000
H	0.583738000	3.299224000	-3.796927000
C	-1.941980000	1.970080000	-0.839937000
C	-3.309582000	2.553952000	-0.593496000
H	-3.348753000	2.915960000	0.437049000
H	-4.046934000	1.751786000	-0.678919000
H	-3.566175000	3.361566000	-1.274886000
C	0.191940000	1.938510000	-2.136118000
O	1.618495000	-1.025147000	0.067204000
O	-0.708178000	-0.990810000	1.480350000
C	1.105110000	-2.474453000	1.879513000
C	-1.053278000	-2.489433000	3.243624000
H	-1.301188000	-1.672421000	3.925873000
H	-1.992887000	-2.838452000	2.807793000
H	-0.583738000	-3.299224000	3.796927000
C	1.941980000	-1.970080000	0.839937000
C	3.309582000	-2.553952000	0.593496000
H	3.348753000	-2.915960000	-0.437049000
H	4.046934000	-1.751786000	0.678919000
H	3.566175000	-3.361566000	1.274886000
C	-0.191940000	-1.938510000	2.136118000
C	-1.580184000	3.546687000	-2.687746000

N	-1.965705000	4.418667000	-3.344232000
C	1.580184000	-3.546687000	2.687746000
N	1.965705000	-4.418667000	3.344232000

19. [Cu((C$_6$H$_5$)COC(NO$_2$)COCH$_3$)$_2$]

Cu	0.000369000	-0.000113000	0.000299000
O	0.856432000	1.649452000	0.465832000
O	0.926656000	-0.011982000	-1.673986000
C	1.876821000	2.194821000	-0.050103000
C	2.462590000	1.729642000	-1.255600000
C	1.896057000	0.704660000	-2.065088000
C	2.335909000	0.439769000	-3.464153000
C	2.712810000	1.464119000	-4.341345000
H	2.740549000	2.493859000	-4.006507000
C	3.030184000	1.169453000	-5.662282000
H	3.311155000	1.969688000	-6.336746000
C	2.988828000	-0.147539000	-6.116085000
H	3.247545000	-0.374781000	-7.144052000
C	2.608329000	-1.170699000	-5.249066000
H	2.570983000	-2.195494000	-5.599944000
C	2.268326000	-0.877661000	-3.934230000
H	1.948161000	-1.659776000	-3.258107000
C	2.430195000	3.368762000	0.719089000
H	2.212830000	4.297532000	0.188605000
H	3.514550000	3.316446000	0.826645000
H	1.953858000	3.378310000	1.697758000
O	-0.855223000	-1.649986000	-0.465023000
C	-1.876084000	-2.194966000	0.050389000
C	-2.462123000	-1.729713000	1.255714000
C	-1.895930000	-0.704556000	2.065190000
O	-0.926457000	0.012125000	1.674315000
C	-2.336175000	-0.439613000	3.464122000
C	-2.712920000	-1.464012000	4.341331000
H	-2.740238000	-2.493788000	4.006562000
C	-3.030703000	-1.169323000	5.662162000
H	-3.311557000	-1.969578000	6.336650000
C	-2.989898000	0.147735000	6.115836000
H	-3.248933000	0.374991000	7.143720000
C	-2.609552000	1.170934000	5.248801000
H	-2.572638000	2.195779000	5.599578000
C	-2.269138000	0.877878000	3.934070000
H	-1.949083000	1.660042000	3.257950000
C	-2.429466000	-3.368739000	-0.719043000
H	-2.210816000	-4.297674000	-0.189327000
H	-3.513936000	-3.317244000	-0.825570000
H	-1.953933000	-3.377452000	-1.698116000
N	3.732445000	2.324616000	-1.679713000
O	4.635080000	1.559428000	-1.991801000
O	3.829939000	3.551308000	-1.687852000

N	-3.731930000	-2.324958000	1.679673000
O	-3.828947000	-3.551686000	1.688161000
O	-4.634922000	-1.560016000	1.991285000

20. [Cu((CH$_3$)COCHCOC(CH$_3$)$_3$)$_2$]

Cu	-0.000001000	0.000028000	-0.001927000
O	-1.336816000	-1.384185000	0.000390000
O	-1.340683000	1.375064000	0.014931000
C	-3.269682000	0.007180000	0.023368000
H	-4.347338000	-0.012700000	0.031952000
C	-3.369838000	2.583237000	0.037435000
C	-2.597436000	-1.226738000	0.011348000
C	-2.606010000	1.243803000	0.024446000
O	1.336824000	1.384190000	-0.005061000
O	1.340694000	-1.374977000	-0.017784000
C	3.269737000	-0.007177000	-0.024386000
H	4.347400000	0.012680000	-0.031515000
C	3.369847000	-2.583241000	-0.036514000
C	2.597464000	1.226721000	-0.014345000
C	2.606022000	-1.243795000	-0.025534000
C	-2.924080000	3.356680000	1.298553000
H	-3.222295000	2.829072000	2.209611000
H	-1.840393000	3.476140000	1.309744000
H	-3.389270000	4.346593000	1.314401000
C	-4.897946000	2.424153000	0.049049000
H	-5.365646000	3.412630000	0.057863000
H	-5.260072000	1.896811000	-0.837463000
H	-5.245659000	1.887817000	0.935936000
C	-2.944568000	3.369436000	-1.222872000
H	-1.861199000	3.489115000	-1.250446000
H	-3.257566000	2.851031000	-2.134252000
H	-3.409896000	4.359410000	-1.221153000
C	4.897970000	-2.424181000	-0.045983000
H	5.365658000	-3.412674000	-0.053485000
H	5.246978000	-1.888406000	-0.932699000
H	5.258800000	-1.896283000	0.840725000
C	2.942701000	-3.368561000	1.223713000
H	1.859290000	-3.488217000	1.249758000
H	3.408011000	-4.358545000	1.223378000
H	3.254350000	-2.849527000	2.135199000
C	2.925931000	-3.357556000	-1.297749000
H	3.225506000	-2.830586000	-2.208728000
H	3.391116000	-4.347493000	-1.312215000
H	1.842260000	-3.476990000	-1.310457000
C	-3.396970000	-2.509655000	0.010817000
H	-3.131195000	-3.095178000	-0.873257000
H	-3.117876000	-3.104247000	0.884671000
H	-4.472856000	-2.335202000	0.019851000
C	3.396983000	2.509658000	-0.014044000

H	3.130437000	3.095791000	0.869401000
H	4.472881000	2.335259000	-0.022071000
H	3.118544000	3.103633000	-0.888524000

21. [Cu(HCOCHCO($C_{10}H_7$))$_2$]

Cu	-0.236877000	0.353758000	-0.272815000
O	0.677671000	1.951604000	0.294654000
O	0.511331000	0.470039000	-2.038165000
C	1.464186000	2.633783000	-0.427625000
C	1.824499000	2.412790000	-1.755960000
H	2.521009000	3.108988000	-2.196691000
C	1.322308000	1.331533000	-2.509099000
C	1.724589000	1.124355000	-3.932061000
C	2.555919000	2.034185000	-4.643915000
H	2.920844000	2.930904000	-4.161133000
C	2.898801000	1.798391000	-5.949735000
H	3.530869000	2.502565000	-6.480538000
C	1.262663000	-0.005211000	-4.581820000
H	0.625974000	-0.690651000	-4.035977000
H	1.910358000	3.506840000	0.067810000
O	-1.151605000	-1.243963000	-0.840369000
C	-1.938459000	-1.925905000	-0.118234000
C	-2.298795000	-1.704898000	1.210091000
H	-2.995744000	-2.400751000	1.650678000
C	-1.796229000	-0.623915000	1.963370000
O	-0.984958000	0.237381000	1.492590000
C	-2.198534000	-0.416773000	3.386325000
C	-3.028728000	-1.327347000	4.098529000
H	-3.392491000	-2.224736000	3.616103000
C	-3.371791000	-1.091521000	5.404299000
H	-4.002843000	-1.796345000	5.935438000
C	-2.913688000	0.065799000	6.087077000
C	-1.737784000	0.713497000	4.035705000
H	-1.101815000	1.399427000	3.489635000
H	-2.384915000	-2.798750000	-0.613789000
C	-2.076504000	0.985869000	5.381021000
C	-3.251312000	0.341668000	7.436033000
C	-1.610172000	2.146616000	6.054152000
C	-2.783633000	1.474337000	8.058399000
C	-1.955234000	2.385608000	7.361557000
H	-3.886244000	-0.356587000	7.971291000
H	-0.975259000	2.841398000	5.514927000
H	-3.048692000	1.674989000	9.090430000
H	-1.594194000	3.274363000	7.866475000
C	1.601081000	-0.277514000	-5.927237000
C	2.439322000	0.641852000	-6.632966000
C	1.133352000	-1.437440000	-6.600785000
C	2.776498000	0.366143000	-7.982065000
C	1.477997000	-1.676279000	-7.908331000

C	2.307397000	-0.765695000	-8.604866000
H	0.497751000	-2.131751000	-6.061764000
H	3.412254000	1.063831000	-8.517084000
H	1.115851000	-2.564397000	-8.413578000
H	2.572088000	-0.966187000	-9.637022000

22. [Cu((CH$_3$)COCHCO(C$_{10}$H$_7$))$_2$]

Cu	0.000000000	-0.000033000	-0.000028000
O	0.231066000	-1.908972000	0.059943000
O	-1.909398000	-0.172445000	-0.060207000
C	-0.687945000	-2.786297000	0.117005000
C	-2.066812000	-2.529514000	0.094365000
H	-2.729530000	-3.376812000	0.168344000
C	-2.609978000	-1.235445000	-0.003998000
C	-4.090175000	-1.024829000	-0.035473000
C	-5.023394000	-2.090091000	-0.175306000
H	-4.673492000	-3.107856000	-0.286440000
C	-6.371993000	-1.845727000	-0.195527000
H	-7.070974000	-2.667691000	-0.309783000
C	-4.566560000	0.268122000	0.072254000
H	-3.850687000	1.075557000	0.165728000
C	-0.197668000	-4.212852000	0.212040000
H	0.425604000	-4.434063000	-0.658500000
H	-1.011880000	-4.935584000	0.265735000
H	0.437191000	-4.314351000	1.096101000
O	-0.231073000	1.908907000	-0.060125000
C	0.687935000	2.786227000	-0.117193000
C	2.066814000	2.529462000	-0.094389000
H	2.729504000	3.376783000	-0.168331000
C	2.609966000	1.235396000	0.004056000
O	1.909363000	0.172412000	0.060274000
C	4.090146000	1.024780000	0.035634000
C	5.023333000	2.090026000	0.175866000
H	4.673419000	3.107755000	0.287331000
C	6.371918000	1.845683000	0.196147000
H	7.070876000	2.667626000	0.310692000
C	6.881590000	0.526378000	0.074399000
C	4.566563000	-0.268126000	-0.072355000
H	3.850736000	-1.075585000	-0.166067000
C	0.197657000	4.212786000	-0.212225000
H	-0.425696000	4.433982000	0.658262000
H	1.011868000	4.935525000	-0.265837000
H	-0.437125000	4.314293000	-1.096340000
C	5.951913000	-0.552091000	-0.060991000
C	8.269764000	0.238474000	0.086537000
C	6.449947000	-1.877108000	-0.179208000
C	8.719000000	-1.055123000	-0.030509000
C	7.800824000	-2.123394000	-0.164462000
H	8.974330000	1.057170000	0.189685000

H	5.741563000	-2.692264000	-0.281325000
H	9.783086000	-1.263118000	-0.020471000
H	8.170480000	-3.138472000	-0.255270000
C	-5.951898000	0.552151000	0.060843000
C	-6.881569000	-0.526342000	-0.074142000
C	-6.449870000	1.877287000	0.178690000
C	-8.269772000	-0.238373000	-0.086194000
C	-7.800728000	2.123552000	0.163975000
C	-8.718963000	1.055246000	0.030455000
H	-5.741462000	2.692442000	0.280551000
H	-8.974349000	-1.057093000	-0.189001000
H	-8.170381000	3.138659000	0.254524000
H	-9.783035000	1.263296000	0.020399000

23. $[Cu((CF_3)COCHCOCF_3)(dmeen)]^+$

Cu	0.933270000	-0.281954000	-0.151870000
O	-0.083824000	1.356544000	-0.233155000
O	-0.681398000	-1.313717000	0.041984000
C	-1.813573000	2.941428000	-0.246260000
C	-1.339876000	1.478420000	-0.136679000
C	-2.276161000	0.458563000	0.040317000
C	-1.866507000	-0.871084000	0.116360000
C	-2.908498000	-1.989036000	0.311068000
N	2.685548000	0.729444000	-0.106973000
N	2.030606000	-1.983623000	-0.318073000
C	3.768521000	-0.272110000	0.106587000
C	3.417307000	-1.545442000	-0.651527000
C	2.696878000	1.867764000	0.871635000
C	3.940654000	2.745396000	0.785638000
H	2.593123000	1.430174000	1.868212000
H	-3.323806000	0.701016000	0.115305000
F	-3.126590000	3.064625000	-0.027619000
F	-1.158919000	3.693945000	0.650778000
F	-1.537701000	3.407651000	-1.471934000
F	-4.156542000	-1.515602000	0.365859000
F	-2.824679000	-2.860096000	-0.705911000
F	-2.648355000	-2.645979000	1.451284000
H	2.807404000	1.137959000	-1.034523000
H	4.738974000	0.105593000	-0.222589000
H	3.836970000	-0.456281000	1.180451000
H	3.450480000	-1.354309000	-1.727080000
H	4.136993000	-2.342182000	-0.436877000
H	1.797597000	2.449925000	0.674764000
H	4.854687000	2.209300000	1.049023000
H	3.839432000	3.581499000	1.480615000
H	4.059654000	3.167846000	-0.216543000
C	1.971224000	-2.692385000	0.989155000
H	2.375071000	-2.063656000	1.782907000
H	0.931496000	-2.920287000	1.217952000

H	2.547373000	-3.622150000	0.946525000
C	1.483154000	-2.866156000	-1.383760000
H	0.455358000	-3.123461000	-1.137396000
H	1.498226000	-2.341682000	-2.340049000
H	2.081171000	-3.779025000	-1.469098000

24. $[Cu((CH_3)COCHCOCH_3)(dmeen)]^+$

Cu	0.064361000	-0.122555000	-0.110204000
O	-0.878880000	1.524382000	-0.327679000
O	-1.526763000	-1.120409000	0.235346000
C	-2.621420000	3.101122000	-0.479945000
C	-2.147333000	1.692635000	-0.245513000
C	-3.069582000	0.679270000	0.046185000
C	-2.723297000	-0.656982000	0.276317000
C	-3.789469000	-1.670711000	0.591112000
N	1.858182000	0.852863000	-0.225392000
N	1.164392000	-1.855343000	-0.164233000
C	2.940388000	-0.140697000	0.007196000
C	2.530367000	-1.470257000	-0.612859000
C	1.934169000	2.060997000	0.655262000
C	3.184535000	2.909146000	0.447104000
H	1.869578000	1.706698000	1.687800000
H	-4.114744000	0.946518000	0.097282000
H	-3.706899000	3.183024000	-0.458356000
H	-2.198710000	3.756212000	0.287230000
H	-2.249438000	3.452352000	-1.445651000
H	-4.782934000	-1.227548000	0.636466000
H	-3.781071000	-2.453944000	-0.171947000
H	-3.563668000	-2.148820000	1.547930000
H	1.930170000	1.184794000	-1.187411000
H	3.894445000	0.188439000	-0.411440000
H	3.074510000	-0.233657000	1.086771000
H	2.501800000	-1.372716000	-1.701273000
H	3.256281000	-2.255046000	-0.373005000
H	1.033220000	2.637847000	0.451541000
H	4.102390000	2.378463000	0.708739000
H	3.129753000	3.798345000	1.078847000
H	3.264324000	3.249980000	-0.589646000
C	1.172161000	-2.422261000	1.208405000
H	1.617022000	-1.716541000	1.910250000
H	0.144000000	-2.611237000	1.511739000
H	1.741866000	-3.357371000	1.238691000
C	0.561954000	-2.838141000	-1.100027000
H	-0.453122000	-3.053175000	-0.774714000
H	0.527628000	-2.415312000	-2.105025000
H	1.149421000	-3.762364000	-1.122274000

25. [Cu(PhCOCHCOCH$_3$)(dmeen)]$^+$

Cu	1.152019000	-0.150125000	-0.090722000
O	-0.723739000	0.188940000	-0.172457000
O	0.926794000	-2.022490000	0.172693000
C	-1.670888000	-0.666788000	-0.015335000
C	-1.454599000	-2.040017000	0.203559000
C	-0.196990000	-2.640106000	0.281150000
C	-0.081772000	-4.125800000	0.494301000
N	1.545721000	1.857193000	-0.078467000
N	3.177029000	-0.400865000	-0.319322000
C	3.016615000	2.041643000	0.042673000
C	3.718910000	0.925712000	-0.720807000
C	0.746559000	2.601348000	0.945625000
C	0.872432000	4.118642000	0.855790000
H	1.071197000	2.240392000	1.925519000
H	-2.314877000	-2.686297000	0.277071000
H	-1.052762000	-4.607117000	0.598810000
H	0.450331000	-4.573307000	-0.349765000
H	0.513604000	-4.318096000	1.390767000
H	1.253368000	2.204252000	-0.992014000
H	3.339746000	3.014303000	-0.336059000
H	3.265528000	2.008541000	1.105189000
H	3.535144000	1.043412000	-1.791931000
H	4.803081000	0.968291000	-0.567455000
H	-0.287242000	2.286422000	0.809734000
H	1.887072000	4.469828000	1.055644000
H	0.214266000	4.582309000	1.593662000
H	0.567149000	4.484158000	-0.129455000
C	3.724875000	-0.851992000	0.984913000
H	3.526445000	-0.110635000	1.759146000
H	3.234124000	-1.782780000	1.263319000
H	4.806501000	-1.011774000	0.917111000
C	3.465549000	-1.423807000	-1.355758000
H	3.003123000	-2.361885000	-1.057717000
H	3.040937000	-1.109810000	-2.310304000
H	4.545704000	-1.562010000	-1.474580000
C	-3.049987000	-0.125888000	-0.103992000
C	-3.271811000	1.062984000	-0.815798000
C	-4.139072000	-0.762119000	0.511076000
C	-4.551479000	1.591419000	-0.925607000
C	-5.416001000	-0.221836000	0.412891000
C	-5.626581000	0.951204000	-0.309907000
H	-2.433916000	1.552443000	-1.294672000
H	-3.989460000	-1.661851000	1.094299000
H	-4.714200000	2.500474000	-1.492524000
H	-6.247111000	-0.714725000	0.902854000
H	-6.624539000	1.365608000	-0.391702000

26. [Cu(PhCOCHCOPh)(dmeen)]$^+$

Cu	0.682263000	-1.181514000	-0.130203000
O	1.148939000	0.663541000	-0.173915000
O	-1.182732000	-0.841987000	0.039053000
C	0.337293000	1.660513000	-0.084755000
C	-1.057136000	1.534565000	0.001581000
C	-1.750009000	0.316385000	0.040850000
N	2.660003000	-1.695271000	-0.025079000
N	0.328275000	-3.184325000	-0.421614000
C	2.759650000	-3.177353000	0.041294000
C	1.638159000	-3.785437000	-0.792239000
C	3.387071000	-0.978338000	1.069329000
C	4.898441000	-1.182620000	1.054387000
H	2.956660000	-1.321018000	2.014445000
H	-1.638944000	2.440212000	0.040051000
H	3.069261000	-1.384196000	-0.905953000
H	3.727463000	-3.539166000	-0.314327000
H	2.671657000	-3.466743000	1.090296000
H	1.809303000	-3.568388000	-1.849823000
H	1.614187000	-4.875396000	-0.680810000
H	3.134092000	0.075485000	0.959663000
H	5.184565000	-2.222338000	1.227696000
H	5.354670000	-0.581373000	1.843730000
H	5.332770000	-0.857796000	0.103957000
C	-0.196723000	-3.745058000	0.847922000
H	0.522762000	-3.604156000	1.654625000
H	-1.112925000	-3.218359000	1.107513000
H	-0.406056000	-4.815384000	0.743286000
C	-0.669497000	-3.387215000	-1.502388000
H	-1.590629000	-2.877905000	-1.228654000
H	-0.295134000	-2.957042000	-2.432317000
H	-0.863077000	-4.454450000	-1.656198000
C	0.969476000	3.004482000	-0.102224000
C	2.218477000	3.162756000	-0.721396000
C	0.360976000	4.120085000	0.492625000
C	2.833172000	4.407748000	-0.762936000
C	0.986344000	5.361330000	0.463530000
C	2.219387000	5.510060000	-0.169072000
H	2.688292000	2.304072000	-1.182946000
H	-0.585292000	4.016437000	1.008673000
H	3.789639000	4.521948000	-1.259422000
H	0.513093000	6.212529000	0.938271000
H	2.700920000	6.480473000	-0.197060000
C	-3.234097000	0.304062000	0.110506000
C	-3.884220000	-0.786320000	0.707348000
C	-4.008670000	1.347779000	-0.418159000
C	-5.270260000	-0.822597000	0.792993000
C	-5.396246000	1.300857000	-0.346151000
C	-6.030166000	0.220233000	0.264480000
H	-3.290932000	-1.591318000	1.120665000

H	-3.532578000	2.182611000	-0.917037000
H	-5.760294000	-1.661728000	1.272652000
H	-5.983589000	2.106317000	-0.770603000
H	-7.111642000	0.189803000	0.326112000

27. [Cu((CF$_3$)COCHCOCH$_3$)(dmeen)]$^+$

Cu	0.680296000	-0.029704000	-0.166478000
O	0.262881000	1.841658000	-0.356282000
O	-1.159480000	-0.492007000	0.060498000
C	-0.904527000	3.882779000	-0.529548000
C	-0.886653000	2.393119000	-0.336983000
C	-2.098225000	1.693428000	-0.139063000
C	-2.138614000	0.323783000	0.048017000
C	-3.485556000	-0.384433000	0.269921000
N	2.679388000	0.337394000	-0.159614000
N	1.164447000	-2.014664000	-0.226561000
C	3.368033000	-0.950320000	0.134318000
C	2.615546000	-2.079823000	-0.556588000
C	3.078867000	1.466531000	0.740750000
C	4.546305000	1.865781000	0.626939000
H	2.834014000	1.161220000	1.761670000
H	-3.024955000	2.245314000	-0.131820000
H	-1.915507000	4.279459000	-0.604357000
H	-0.397505000	4.358219000	0.315566000
H	-0.341564000	4.139244000	-1.430051000
F	-4.522547000	0.460971000	0.240722000
F	-3.670596000	-1.313052000	-0.683720000
F	-3.478804000	-1.005641000	1.460273000
H	2.923174000	0.617973000	-1.110034000
H	4.409874000	-0.937628000	-0.193779000
H	3.368730000	-1.081013000	1.218287000
H	2.710774000	-1.974736000	-1.640381000
H	3.033949000	-3.054446000	-0.283524000
H	2.427349000	2.300968000	0.484973000
H	5.225006000	1.072160000	0.945951000
H	4.735914000	2.732951000	1.262969000
H	4.801947000	2.150769000	-0.398103000
C	0.873734000	-2.588292000	1.114468000
H	1.463919000	-2.084975000	1.880685000
H	-0.182150000	-2.442285000	1.335373000
H	1.107685000	-3.657775000	1.133603000
C	0.355225000	-2.724804000	-1.252284000
H	-0.699232000	-2.608759000	-1.012268000
H	0.544132000	-2.290274000	-2.234912000
H	0.614520000	-3.788281000	-1.277654000

28. [Cu((CF$_3$)COCHCOPh)(dmeen)]$^+$

Cu	-1.154543000	-0.412178000	-0.135112000
O	0.740310000	-0.736696000	-0.182297000
O	-0.916588000	1.468215000	0.039002000
C	1.694473000	0.111042000	-0.079589000
C	1.474567000	1.506888000	0.064243000
C	0.213728000	2.062114000	0.107874000
C	0.051637000	3.583783000	0.244070000
N	-1.568889000	-2.400352000	-0.029405000
N	-3.164468000	-0.129252000	-0.384170000
C	-3.043524000	-2.547230000	0.121886000
C	-3.734783000	-1.467522000	-0.700998000
C	-0.770750000	-3.109859000	1.021399000
C	-0.929241000	-4.626530000	1.007793000
H	-1.074894000	-2.693938000	1.985783000
H	2.319095000	2.173864000	0.105483000
H	-1.299375000	-2.800859000	-0.928170000
H	-3.387711000	-3.535665000	-0.191010000
H	-3.275602000	-2.442506000	1.183509000
H	-3.565635000	-1.652517000	-1.764942000
H	-4.817231000	-1.480452000	-0.533795000
H	0.267867000	-2.824578000	0.858517000
H	-1.947496000	-4.945452000	1.239690000
H	-0.269679000	-5.066801000	1.758624000
H	-0.646591000	-5.046759000	0.037759000
C	-3.704754000	0.420057000	0.887262000
H	-3.529067000	-0.275401000	1.708326000
H	-3.193071000	1.355221000	1.107724000
H	-4.781243000	0.601409000	0.801159000
C	-3.423581000	0.833764000	-1.486804000
H	-2.939717000	1.778593000	-1.249632000
H	-3.006355000	0.447288000	-2.417746000
H	-4.499608000	0.990007000	-1.616588000
C	3.069684000	-0.427910000	-0.142951000
C	3.280407000	-1.693775000	-0.714352000
C	4.171658000	0.284487000	0.358503000
C	4.559432000	-2.225820000	-0.795659000
C	5.448391000	-0.259037000	0.289818000
C	5.646148000	-1.510443000	-0.291583000
H	2.433782000	-2.239422000	-1.109377000
H	4.036206000	1.248974000	0.830462000
H	4.714213000	-3.195387000	-1.253770000
H	6.289571000	0.293201000	0.690751000
H	6.644559000	-1.927364000	-0.351850000
F	1.225815000	4.226673000	0.302040000
F	-0.639068000	3.869092000	1.360604000
F	-0.638693000	4.064539000	-0.804503000

29. [Cu((CF$_3$)COCHCOC$_4$H$_3$S)(dmeen)]$^+$

Cu	-1.096529000	-0.429171000	-0.126661000

O	0.815200000	-0.641571000	-0.137841000
O	-0.976656000	1.461466000	0.058896000
C	1.711054000	0.274760000	-0.055928000
C	1.406616000	1.655824000	0.094734000
C	0.114565000	2.126884000	0.141388000
C	-0.144761000	3.631457000	0.302755000
N	-1.392879000	-2.439191000	-0.046997000
N	-3.112619000	-0.264948000	-0.422705000
C	-2.859404000	-2.677715000	0.059261000
C	-3.592262000	-1.630914000	-0.770004000
C	-0.584550000	-3.111367000	1.020232000
C	-0.655000000	-4.634454000	0.990434000
H	-0.937936000	-2.722350000	1.979038000
H	-1.071954000	-2.811912000	-0.940930000
H	-3.133247000	-3.680804000	-0.275616000
H	-3.127524000	-2.602147000	1.114850000
H	-3.383799000	-1.791474000	-1.830910000
H	-4.676089000	-1.710994000	-0.632833000
H	0.440271000	-2.767345000	0.887764000
H	-1.659152000	-5.013488000	1.191320000
H	0.007884000	-5.043001000	1.756135000
H	-0.321626000	-5.028232000	0.025506000
C	-3.714836000	0.233543000	0.841719000
H	-3.516499000	-0.460939000	1.658433000
H	-3.265840000	1.194795000	1.085412000
H	-4.798051000	0.350730000	0.731895000
C	-3.404070000	0.695085000	-1.519792000
H	-2.984525000	1.664227000	-1.259656000
H	-2.941883000	0.347096000	-2.444643000
H	-4.484335000	0.786734000	-1.673850000
F	0.986696000	4.347035000	0.374355000
F	-0.854266000	3.855432000	1.421292000
F	-0.861809000	4.085636000	-0.740084000
C	4.258877000	0.576871000	-0.030383000
C	3.091221000	-0.159740000	-0.125477000
S	3.447633000	-1.855836000	-0.365809000
C	5.137847000	-1.541597000	-0.330238000
C	5.423012000	-0.211290000	-0.146406000
H	4.281509000	1.647180000	0.120644000
H	5.835310000	-2.357172000	-0.450457000
H	6.428962000	0.181516000	-0.097389000
H	2.210412000	2.369736000	0.167047000

30. [Cu((CF$_3$)COCHCOCF$_3$)(dmeen)]$^+$

Cu	0.933270000	-0.281954000	-0.151870000
O	-0.083824000	1.356544000	-0.233155000
O	-0.681398000	-1.313717000	0.041984000
C	-1.813573000	2.941428000	-0.246260000
C	-1.339876000	1.478420000	-0.136679000

C	-2.276161000	0.458563000	0.040317000
C	-1.866507000	-0.871084000	0.116360000
C	-2.908498000	-1.989036000	0.311068000
N	2.685548000	0.729444000	-0.106973000
N	2.030606000	-1.983623000	-0.318073000
C	3.768521000	-0.272110000	0.106587000
C	3.417307000	-1.545442000	-0.651527000
C	2.696878000	1.867764000	0.871635000
C	3.940654000	2.745396000	0.785638000
H	2.593123000	1.430174000	1.868212000
H	-3.323806000	0.701016000	0.115305000
F	-3.126590000	3.064625000	-0.027619000
F	-1.158919000	3.693945000	0.650778000
F	-1.537701000	3.407651000	-1.471934000
F	-4.156542000	-1.515602000	0.365859000
F	-2.824679000	-2.860096000	-0.705911000
F	-2.648355000	-2.645979000	1.451284000
H	2.807404000	1.137959000	-1.034523000
H	4.738974000	0.105593000	-0.222589000
H	3.836970000	-0.456281000	1.180451000
H	3.450480000	-1.354309000	-1.727080000
H	4.136993000	-2.342182000	-0.436877000
H	1.797597000	2.449925000	0.674764000
H	4.854687000	2.209300000	1.049023000
H	3.839432000	3.581499000	1.480615000
H	4.059654000	3.167846000	-0.216543000
C	1.971224000	-2.692385000	0.989155000
H	2.375071000	-2.063656000	1.782907000
H	0.931496000	-2.920287000	1.217952000
H	2.547373000	-3.622150000	0.946525000
C	1.483154000	-2.866156000	-1.383760000
H	0.455358000	-3.123461000	-1.137396000
H	1.498226000	-2.341682000	-2.340049000
H	2.081171000	-3.779025000	-1.469098000
